# Serum concentration of iron as predictor of cancer risk among BRCA1 mutation carriers

**DOI:** 10.1186/1897-4287-10-S3-A21

**Published:** 2012-04-20

**Authors:** Grzegorz Sukiennicki, Magdalena Muszyńska, Tomasz Huzarski, Jacek Gronwald, Cezary Cybulski, Tadeusz Dębniak, Aleksandra Tołoczko-Grabarek, Oleg Ashuryk, Anna Jakubowska, Antoni Morawski, Jan Lubiński

**Affiliations:** 1 Read-Gene SA and Pomeranian Medical University, Szczecin, Poland

## 

The aim of the study is identification of correlations between the serum concentrations of iron and the risk of breast and/or ovarian cancer among female BRCA1 mutations carriers.

The subjects selected for the trial were Polish women, positive for at least one of three founder mutations in BRCA1 gene dominating in Poland (5382insC, C61G, 4153delA). Persons with detected tumor were considered as cases and the others were considered as controls. One case and two controls were paired regarding many criteria (e.g. age, family cancer history, cigarettes smoking) to achieve the maximum of similarity between them.

The proportion of cases and control in the first quartile was taken as a reference to calculate the odds ratio, confidence interval and p-value of the multivariate conditional logistic regression.

The iron was quantitatively measured by ICP-MS (Inductively Coupled Plasma Mass Spectrometry), (model Elan DRC-e 6100 th, PerkinElmer).

This study shows that concentration levels of iron in blood serum are a strong factors associated with an additionally increased risk of breast and ovarian cancer among BRCA1 mutation carriers.

For iron concentration, all quartiles above the first one had a decreased risk of breast or ovarian cancer. The results are shown in Table 1.

**Table 1:** Iron concentration in each quartile

Fe (µg/l)	Cases (n=99)	Controls (n=198)	OR	p-value
370,95 – 977,64[	34 (34,3%)	40 (20,2%)	1,000	-
[977,64 – 1262,78[	24 (24,2 %)	50 (25,3%)	0,565	0.01229
[1262,78 – 1571,11[	24 (24,2%)	50 (25,3%)	0,565	0.13885
[1571,11 – 4756,15	17 (17,17%)	58 (29,3%)	0,345	0.00823

Similarly, high ratios of iron to selenium were significantly associated with disease protection which is shown in Table 2.

**Table 2:** Ratios between iron and selenium

Fe/Se	Cases (n=99)	Controls (n=198)	OR	p-value
0.86-12.78	34 (34,3%)	40 (20,2%)	1,000	-

12.78-15.84	25 (25,3%)	49 (24,7%)	0,600	0.094608

15.84-19.72	18 (18,2%)	56 (28,3%)	0,378	0.006507

19.72-59.3	22 (22,2%)	53 (26,8%)	0,488	0.007813

